# Feedback inhibition of CREB signaling by p38 MAPK contributes to the negative regulation of steroidogenesis

**DOI:** 10.1186/s12958-017-0239-4

**Published:** 2017-03-16

**Authors:** Jiaxin Li, Qian Zhou, Zhuang Ma, Meina Wang, Wen-Jun Shen, Salman Azhar, Zhigang Guo, Zhigang Hu

**Affiliations:** 1 Jiangsu Key Laboratory for Molecular and Medical Biotechnology, College of Life Sciences, Nanjing Normal University, 1 WenYuan Road, Nanjing, 210023 China; 2 Geriatric Research, Education and Clinical Center, VA Palo Alto Health Care System, Palo Alto, CA 94304 USA; 3 Stanford University School of Medicine, Palo Alto, CA 94304 USA

**Keywords:** Steroidogenesis, p38 MAPK, CREB, Steroids intermediates, Feedback regulation

## Abstract

**Background:**

Steroidogenesis is a complex, multi-steps biological process in which, cholesterol precursor is converted to steroids in a tissue specific and tropic hormone dependent manner. Given that steroidogenesis is achieved by coordinated functioning of multiple tissue specific enzymes, many steroids intermediates/metabolites are generated during this process. Both the steroid products as well as major lipoprotein cholesterol donor, high-density lipoprotein 3 (hHDL_3_) have the potential to negatively regulate steroidogenesis via increased oxidative stress/reactive oxygen species (ROS) generation.

**Methods:**

In the current study, we examined the effects of treatment of a mouse model of steroidogenesis, Y1-BS1 adrenocortical tumor cells with pregnenolone, 22(R)-Hydroxycholesterol [22(R)-diol] or hHDL_3_ on ROS production, phosphorylation status of p38 MAPK and cAMP response element-binding protein (CREB), CREB transcriptional activity and mRNA expression of StAR, CPY11A1/P450scc and antioxidant enzymes, superoxide dismutases [Cu,ZnSOD (SOD1), MnSOD (SOD2)], catalase (CAT) and glutathione peroxidase 1 (GPX1). We also detected the steroid product in p38 MAPK inhibitor treated Y1 cells by HPLC-MS / MS.

**Results:**

Treatment of Y1 cells with H_2_O_2_ greatly enhanced the phosphorylation of both p38 MAPK and CREB protein. Likewise, treatment of cells with pregnenolone, 22(R) diol or hHDL_3_ increased ROS production measured with the oxidation-sensitive fluorescent probe 2′,7′-Dichlorofluorescin diacetate (DCFH-DA). Under identical experimental conditions, treatment of cells with these agents also increased the phosphorylation of p38 MAPK and CREB. This increased CREB phosphorylation however, was associated with its decreased transcriptional activity. The stimulatory effects of pregnenolone, 22(R)-diol and hHDL_3_ on CREB phosphorylation was abolished by a specific p38 MAPK inhibitor, SB203580. Pregnenolone, and 22(R) diol but not hHDL_3_ upregulated the mRNA expression of SOD1, SOD2 and GPX1, while down-regulated the mRNA levels of StAR and CYP11A1. The p38 inhibitor SB203580 could increase the steroid production in HDL3, 22(R)-diol or pregnenolone treated cells.

**Conclusion:**

Our data demonstrate induction of a ROS/p38 MAPK -mediated feedback inhibitory pathway by oxy-cholesterol and steroid intermediates and products attenuates steroidogenesis via inhibition of CREB transcriptional activity.

## Background

Steroidogenesis is a multi-step process by which the cholesterol is converted to parent steroid, pregnenolone, which is further metabolized into other steroids in a tissue specific manner [1, 2]. The cholesterol required for steroid hormone synthesis can be theoretically obtained from several different potential sources including de novo synthesis from acetate, cholesteryl esters stored in the form of lipid droplets or can be obtained from circulating lipoproteins via low-density lipoprotein (LDL) receptor/endocytic pathway or SR-BI (for high-density lipoprotein or HDL)/selective uptake pathway [3, 4]. steroidogenic process is subjected to a dual regulation—acute and chronic regulation [5], although both are under the control of tissue-specific tropic hormone. Adrenocorticotropin hormone (ACTH) increases glucocorticoid (cortisol in humans and corticosterone in rodents) synthesis in adrenal fasciculata cells, ACTH, K^+^ or angiotensin II (AngII) control mineralocorticoid (aldosterone) synthesis in adrenal glomerulosa cells, follicle-stimulating hormone (FSH) controls the female sex steroid (progesterone and estrogen) synthesis in ovarian granulosa cells, whereas luteinizing hormone (LH) regulates progesterone synthesis in luteinized ovarian granulosa-luteal cells, androgen production in ovarian theca-interstitial cells and testosterone synthesis in testicular Leydig cells [2].

ROS, such as H_2_O_2_ and superoxide anion (O_2_^●–^) are produced by the mitochondrial electron transport chain as a byproduct of oxidative phosphorylation [6]. In steroidogenic cells, there is a secondary source for ROS production. During steroidogenesis, ROS is produced by cytochrome P450 enzymes catalyzing the steroid hydroxylation steps, particularly by CYP11B1 and to a lesser extent by CYP11A1 [7]. Moreover, tropic hormones, ACTH and LH not only stimulate steroid synthesis in steroidogenic cells of their target tissues but also promote ROS production, which in turn can cause DNA damage, protein oxidation and membrane lipid peroxidation in steroidogenic cells [8, 9]. We have previously reported that age-related decline in steroid synthesis is caused by excessive oxidative damage to cellular machinery involved in steroidogenesis. In addition, we demonstrated that aging leads to a significant reduction in enzymatic and non-enzymatic antioxidant systems and increased membrane lipid peroxidation in steroidogenic cells of adrenal gland and testis [10].

Extensive evidence now suggests that low (physiological) but not high concentrations of ROS such as H_2_O_2_ contribute to the regulation of multiple cellular signaling pathways [11–13]. Given this, we considered the possibility that ROS generated during steroidogenesis may have a negative feedback effect on steroid hormone synthesis. Indeed, there is evidence that ROS such as H_2_O_2_ interferes with the normal transport of mobilized cytoplasmic cholesterol to and within the mitochondria for side chain cleavage in steroidogenic cells [14–16]. Current evidence suggests that one mechanism by which ROS may influence cellular signaling pathways, is via increased phosphorylation and activation of oxidant sensitive p38 MAPKs [17–19]. p38 MAPKs are activated by a wide variety of cellular stresses, such as cytokines, ultraviolet irradiation, heat shock, and osmotic shock, and in turn regulate a number of metabolic processes including cell proliferation, differentiation, apoptosis and other numbers of different biological effects [20–22]. Our own studies have shown that oxidant mediated activation of p38 MAPK inhibits steroidogenesis via down-regulation of StAR gene transcription [22, 23].

Besides oxidants, there is ample evidence to suggest that tropic hormones (e.g., FSH and ACTH) themselves can stimulate the phosphorylation and activation of p38 MAPK. The hormonal activation of p38 MAPK, is presumably required to terminate the hormone-stimulated acute steroidogenesis. When considered in this context, it is likely that p38 MAPK serves as a negative regulator of steroidogenesis via a feedback dependent mechanism. In the current study, we sought to determine whether the steroid intermediates or precursors can stimulate p38 MAPK phosphorylation/activation. We provide evidence that the steroid substrate/precursors and intermediates, HDL_3_, 22(R)-diol and pregnenolone, can promote the phosphorylation and activation of p38 MAPK. In addition, phosphorylation and activity of CREB protein, a key transcription factor involved in the transcriptional regulation of steroidogenic enzymes, is also regulated by these metabolites. The mRNA expression of StAR and CYP11A1 (P450scc), which mediate the first steps in steroidogenesis, were detected and regulated by these steroid precursors and intermediates. Finally, we demonstrate that expression of antioxidant enzymes, SODs and GPX is also subjected to regulation by the steroid substrate/precursors and intermediates. In summary, this study provides first evidence that treatment of a model rodent adrenal cell line with steroid precursors/ metabolites promote ROS production, stimulate (activate) p38 MAPK and CREB phosphorylation and changed gene transcription of StAR, CYP11A1 and three major antioxidant enzymes. The ROS/p38 MAPK-mediated increased phosphorylation of CREB, however, is associated with a reduction in its transcriptional activity. This attenuation in CREB transcriptional activity in turn results in inhibition of mRNA expression of key steroidogenic enzymes/proteins such as StAR via a feedback mechanism.

## Methods

### Reagents and antibodies

22(R)-Hydroxycholesterol [22(R)-diol], 21-Acetoxypregnenolone and p38 MAP kinase inhibitor (SB 203580) were obtained from Sigma–Aldrich (St. Louis, MO). Promega Dual-Luciferase Reporter Kit (E1980) was purchased from Promega Corporation (Madison, WI). pCRE-Luc cis-reporter plasmid and pLuc-MCS vector were obtained from Agilent Technologies (Santa Clara, CA, USA). 20α-hydroxyprogesterone was purchased from Chemsky International Co., Ltd. (Shanghai, China). Anti-phospho-CREB (Ser133), anti-CREB, anti-p38 MAPK, and anti-phospho-p38 MAPK (Thr180/Tyr182) antibodies were supplied by Cell Signaling Technology, Inc. (Danvers, MA, USA). Anti-GAPDH was purchased from Santa Cruz Biotechnology (Santa Cruz, CA, USA). IRDye® 800CW Goat anti-Mouse IgG (H + L) and IRDye® 800CW Goat anti-rabbit IgG (H + L) were purchased from LI-COR Biosciences (Lincoln, NE, USA). SYBR Green Master Mix was supplied by TAKARA Biotechnology (Dalian) Co. LTD (Dalian, China). Lipofectamine® 2000 was obtained from Invitrogen (Life Technologies, Grand Island, NY, USA). Tissue culture supplies were from Life Technologies through its Gibco Cell Culture Media Division (Grand Island, NY, USA).

### Cell culture and treatment

SR-BI enriched Y1-BS1 cells were initially kindly supplied by late Dr David Williams (State University at Stony Brook, Stony Brook, NY, USA). Y1-BS1 cells, which is response to hormone and lipoprotein with increased steroidogenesis, is a stable subclone of the Y1 mouse adrenocortical tumor cell line isolated by Yasumura et al. 1966 [24, 25]. Y1-BS1 has been proven to be robust in steroidogenesis and retains responsiveness to hormone [24]. In contrast to mouse adrenocortical cells/adrenal gland that produce corticosterone as the major steroid product, cultured Y1-BS1 cells, both under basal conditions and in response to trophic hormone stimulation, synthesize and secrete 20α-hydroxy-∆4-pregnene-3-one (20α-hydroxyprogesterone) and 11β,20α-dihydroxy-∆4-pregnene-3-one (11β, 20α-hydroxyprogesterone) [26]. Y1-BS1 cells also secrete small quantities of progesterone. Y1-BS1 cells were cultured in F-12K medium supplemented with 10% fetal bovine serum (FBS), and 100 unit/ml penicillin and 100 μg/ml streptomycin. Cell cultures were maintained at 37 °C in a humidified incubator in the presence of 5% CO2/95% air. When required, cells were incubated with H_2_O_2_ (100 nM) or Bt2cAMP (2.5 mM) for an appropriate time [27]. For other treatment, cells were plated and cultured in 6-well or 12-well plates with 10% bovine (b) lipoprotein-deficient serum (LPDS), and subsequently treated with human (h) apoE-free high density lipoproteins (hHDL_3_, 30 μg/ml), 22(R)-diol (10 μM) or pregnenolone (10 μM) for varying incubation time. hHDL_3_ and bLPDS were isolated as previously described [28, 29].

### Measurement of intracellular reactive oxygen species (ROS) production

The levels of intracellular ROS were quantified using the Reactive Oxygen Species Assay Kit purchased from Beyotime Biotechnology (Shanghai, China). In brief, triplicate dishes of cultured Y1-BS1 cells (5X10^5^) cells/well were pre-loaded with 10 μM DCFH-DA (2,7-Dichlorodi -hydrofluorescein diacetate) for 30 min. Subsequently, dishes were washed and incubated with HDL3 (30 μg protein/ml), 22(R)-diol (10 μM) or pregnenolone (10 μM) for indicated time. At the end of incubation, the culture medium was removed from dishes and cells were immediately lysed and centrifuged. DCFH-DA fluorescence of the cell lysates was measured using Tecan Infinite® 200 Pro Microplate Reader with excitation and emission wavelengths of 480 and 525 nm respectively.

### Luciferase assays

Luciferase assays were carried out using cell extracts from transfected Y1-BS1 cells. Groups of cultured dishes with 60% confluent Y1-BS1 cells were transfected with 1 μg pCRE- luc plasmid or pLuc-MCS vector per well in a 24-well plate using Lipofectamine® 2000 as a transfection reagent (Life Technologies, Grand Island, NY, USA). Twenty-four hours after transfection, the cells were re-cultured in F-12K medium supplemented with 10% LPDS overnight followed by treatment with HDL_3_, 22(R)-diol or pregnenolone for an additional 5 h. Cells were harvested, lysed and cell extracts luciferase assayed with dual-luciferase reporter assay system (Promega) according to the manufacturer’s protocol and light signal (bioluminescence) was quantified using a Tecan Infinite® 200 Pro luminometer. Renilla luciferase was used as a normalization control. The results are expressed as relative luciferase activity (ratio of Firefly/Renilla luciferase activity), and data shown are the mean (±SD) of triplicate values obtained from a representative experiment that was independently repeated for at least three times.

### RNA isolation and quantitative PCR

Total RNA was extracted from Y1-BS1 cells using a miRNeasy mini kit (Qiagen Inc., Valencia, CA) following manufacturer’s instructions. The isolated RNA was transcribed to first strand cDNA at 42 °C for 1 h in an incubation medium containing 2 μg of total RNA and superscript II reverse transcriptase (Life Technologies, Grand Island, NY). Amplification of cDNAs was performed with an ABI StepOneplus system according to manufacturer’s instructions. Each sample contained 2 μl cDNA (1:10 dilution of the original cDNA), 500 nM each of sense and antisense primer,10 μl 2x SYBER Green premix and 0.4 μl Rox (qPCR kit, cat. no. RR420A; TAKARA Biotechnology, Dalian, China) in a final volume of 20 μl. 36B4 was used as an internal control. All primer sequences used for qPCR are presented in Table 1.

**Table 1:** Primers used for quantitative real-time PCR

Primers for qPCR	
Mouse StAR	5′-CGGAGCAGAGTGGTGTCATC-3′-F 5′-TGAGTTTAGTCTTGGAGGGACTTC-3′-R
Mouse CYP11A1	5′-ACTGTGAACTGAAGGCTGG-3′-F 5′-GGGAAAGAGGGAAAGAGGATG-3′-R
Mouse SOD1	5′-AAGACTGGAAATGCTGGGAG-3′-F 5′-GGTTTGAGGGTAGCAGATGAG-3′-R
Mouse SOD2	5′-TGCTCTAATCAGGACCCATTG-3′-F 5′-CATTCTCCCAGTTGATTACATTCC-3′-R
Mouse CAT	5′-TCACCTGTAATCAACGCTGG-3′-F 5′-AGCCCTAACCTTTCATTTCCC-3′-R
Mouse GPX1	5′-CAGGAGAATGGCAAGAATGAAG-3′-F 5′-GAAGGTAAAGAGCGGGTGAG-3′-R
Mouse 36B4	5′-TTTGGGCATCACCACGAAAA-3′-F 5′-GGACACCCTCCAGAAAGCGA-3′-R

### Western blot analysis

For Western blotting, Y1-BS1 cell lysates were mixed with equal volumes of 5 × Laemmli sample buffer [120 mM Tris-HCl, pH 6.8, 2% SDS (w/v), 10% sucrose (w/v), and 1% 2-mercaptoethanol] and subjected to 12% SDS-PAGE. For each sample, a constant amount of protein (10–20 μg) was loaded on the gel. Protein markers were also loaded on the gel. After electrophoretic separation, the proteins were transferred to Immobilon PVDF membranes (Millipore Corp., Bedford, MA) using standard techniques. The protein blots were incubated with first antibody for 2 h at room temperature, then probed with IRDye infrared secondary mouse or rabbit anti-rabbit IgG and visualized using the odyssey® infrared imaging system (LI-COR Biosciences, Lincoln, NE, USA).

### Hormone analysis

Y1-BS1 cells were pre-loaded into 12-well plate (5X10^5^ cells/well) and then cultured in F-12K medium supplemented with 10% LPDS ± p38 MAPK inhibitor SB203580 overnight. Subsequently, dishes were washed and incubated with hHDL_3_ (30 μg protein/ml), 22(R)-diol (10 μM) or pregnenolone (10 μM) for 5 h. The medium samples were collected for hormone analysis using isotope dilution high performance liquid chromatography-tandem mass spectrometry (ID-HPLC-MS / MS). HPLC-MS / MS was performed by Shanghai Dian Medical Testing Institute (Shanghai, China) as previously escribed [30].

### Statistical analysis

Data are expressed as mean ± SEM for at least three independent experiments. Statistical analyses were performed using ANOVA followed by the Bonferroni’s post-test using GraphPad Prism Software, Prism 6 (GraphPad Software, Inc., San Diego, CA, USA). A statistical difference of *p* < 0.05 was considered significant.

## Results

### H_2_O_2_ stimulates the phosphorylation of p38 MAPK and cAMP responsive CREB transcription factor

**Fig. 1:**
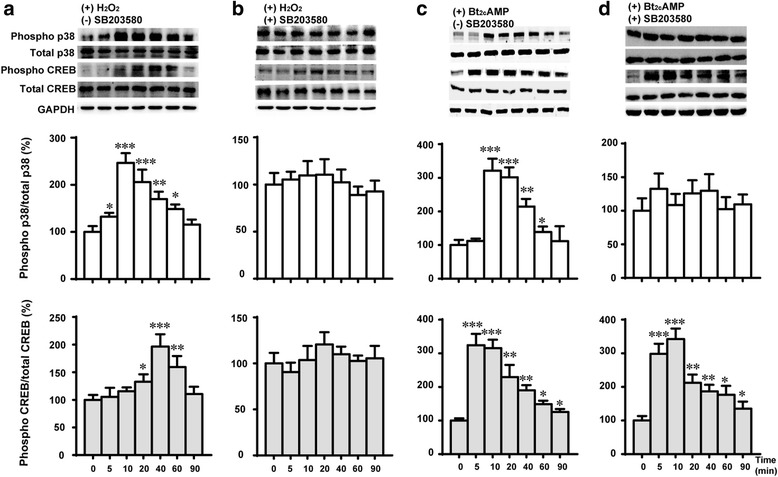
H_2_O_2_ and Bt_2_cAMP treatment increases phosphorylation of p38 MAPK and CREB in time course manner. Y1-BS1 cells were pre-treated without (**a**, **c**) or with (**b**, **d**) p38 MAPK inhibitor SB203580 overnight and then cultured in medium ± H_2_O_2_ (100 nM) or Bt_2_cAMP (2.5 mM) for different times. The cell lysates were subjected to SDS–PAGE followed by western blotting analysis. Phospo-specific and total p38 MAPK and CREB were detected and GAPDH used as loading control. Results are summary of three independent experiments with triplicates in each experiment. * *p* < 0.05, ** *p* < 0.01, *** *p* < 0.001

Increasing evidence suggests that ROS such as •O_2_^-^, •OH and H_2_O_2_, promote the phosphorylation and activation of MAPK, among which p38 MAPK is highly responsive to oxidant stress which has important roles in cell signaling and homeostasis [31–34]. We previously demonstrated that treatment of Y1-BS1 cells with superoxide, H_2_O_2_ or a lipid peroxidation product, 4-hydroxy-2-nonenal (HNE) reciprocally inhibited steroid production and increased the phosphorylation and activation of p38 MAPK [23]. CREB is a key transcription factor that plays a pivotal role in the regulation of steroidogenic enzymes at their gene transcription level. It is stimulated by a number of agents including cAMP, growth factors and UV exposure [35–37]. The results presented in Fig. 1a and b demonstrate that treatment of Y1-BS1 cells with 100 nM H_2_O_2_ for 5 min caused a robust phosphorylation of p38 MAPK and a modest but significant stimulation of phosphorylated form of CREB for 20 min, thereafter the phosphorylation of p38 and CREB decreased. Pretreatment of cells with a specific p38 MAPK inhibitor, SB203580 abolished the stimulatory effects of H_2_O_2_ on p38 MAPK and CREB phosphorylation. These results not only complement the previously published studies from our laboratory [22] but also unequivocally establish that CREB is a downstream critical target of p38 MAPK in steroidogenic cells. Meanwhile, we detected that Bt_2_cAMP could stimulated both the phosphorylation of p38 MAPK and CREB (Fig. 1c). While SB203580 abolished the stimulatory phosphorylation of p38 MAPK by Bt_2_cAMP, phosphorylation of CREB was not diminished (Fig.1d). These results are consistent with the reported finding that cAMP stimulated the phosphorylation of CREB through PKA pathway [36, 38].

### Steroid intermediates, 22(R)-diol and pregnenolone and circulating cholesterol carrier hHDL_3_ promote intracellular ROS production

**Fig. 2:**
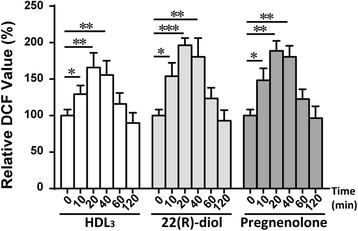
**S**teroid intermediates stimulate reactive oxygen species (ROS) production and oxidative stress in Y1-BS1 cells. Y1-BS1 cells were pre-loaded with 10 μM DCFH-DA for 30 min, washed and incubated with HDL_3_ (30 μg/ml), 22(R)-diol (10 μM) or pregnenolone (10 μM) for different time point. At the end of incubation, the media were removed and cells were immediately lysed and centrifuged. DCFH-DA fluorescence of the cell lysates was measured using a microplate reader with excitation and emission wavelengths of 480 and 525 nm respectively. Results are summary of three independent experiments with triplicates in each experiment. * *p* < 0.05, ** *p* < 0.01, ***p < 0.001

Steroidogenesis being a multi-step process results in generation and/or accumulation of a number of intermediary metabolites such as 22(R)-diol and pregnenolone, which could potentially further enhance the intracellular ROS production. This possibility is in line with the earlier evidence showing that the steroid intermediates, testosterone, testosterone precursors or analogs e.g., epitestosterone and 17a-methyltestosterone, can cause oxygen tension sensitive decrement in P450 hydroxylase activity [39]. In addition, the cholesterol carrier, hHDL_3_ may also directly or indirectly influence the ROS production, especially since HDL is known to participate in cellular signaling [40–42] and is the predominant form of circulating lipoprotein in rodent and a major source of cholesterol for steroidogenesis. Here, we tested the ability of HDL_3_, 22(R)-diol and pregnenolone to modulate ROS production in Y1-BS1 cells. For these studies, Y1-BS1 cells were cultured in regular F-12K medium containing 10% FBS for a brief period of time to allow the cells to attach to the plate and then culture medium was replaced with a fresh medium containing 10% LPDS and dishes incubated overnight. Subsequently, the cells were treated ± HDL_3_, ±22(R)-diol or ± pregnenolone in the presence of 10% LPDS for various time points. At the end of incubation, intracellular ROS generation was evaluated using DCFH-DA assay. The results depicted in Fig. 2 demonstrate that HDL_3_, 22(R)-diol or and pregnenolone, all stimulated ROS generation with a biphasic response; an initial 10 min rapid response and a gradually declining slow phase during the next 60 min (Fig. 2).

### Treatment of Y1-BS1 cells with steroid linked intermediates/metabolites and hHDL_3_ results in increased phosphorylation of p38 MAPK

**Fig. 3:**
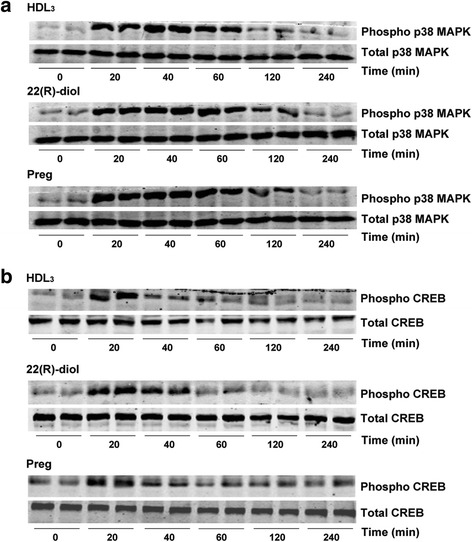
Steroid intermediates promote phosphorylation of p38 MAPK (**a**) and CREB (**b**) in a time dependent manner. Y1-BS1 cells were incubated with HDL_3_ (30 μg/ml), 22(R)-diol (10 μM) or pregnenolone (10 μM) for different time point. At the end of incubation, cells were harvested and lysed for SDS–PAGE followed by western blotting analysis using Phospo-specific and total p38 MAPK and CREB antibodies. Data shown are representative of three independent experiments

**Fig. 4:**
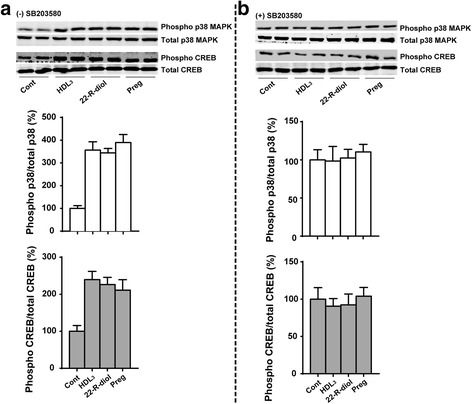
Inhibition of p38 MAPK blocked the phosphorylation of CREB by steroid intermediates. Y1-BS1 cells were pre-treated without (**a**) or with (**b**) p38 MAPK inhibitor (10 μM) overnight. Cells were then incubated with HDL_3_ (30 μg/ml), 22(R)-diol (10 μM) or pregnenolone (10 μM) for 20 min. At the end of the incubation, cell lysates were subjected to SDS–PAGE followed by western blotting analysis. Data shown are representative of three independent experiments

We next tested the ability of HDL_3_, 22(R)-diol and pregnenolone to promote the phosphorylation of p38 MAPK. Treatment of Y1-BS1 with hHDL_3_ (30 μg protein/ml), 22(R)-diol (10 μM) or pregnenolone (10 μM) increased p38 MAPK phosphorylation in a time dependent manner (Fig. 3a). In each case, the maximum stimulatory effect of the agent was noted between 20 and 40 min after exposure and thereafter phosphorylation levels of p38 MAPK began to decline reaching to the basal levels at the end of 240 min of incubation. The total p38 MAPK levels, however, were not impacted by treatment of the cells with any of the three agents (Fig. 3a). We next evaluated whether hHDL_3_ and/or steroid metabolites can modulate CREB phosphorylation via p38 MAPK. CREB is one of the best understood phosphorylation dependent transcription factors [43–45]. It activates transcription of the target genes in response to a diverse array of stimuli including peptide hormones, growth factors and neuronal activity. Figure 3b shows that HDL_3_, 22(R)-diol or pregnenolone treatment, like H_2_O_2_, increased the phosphorylation of CREB with maximal stimulation occurring around 20 min. These results led us to conclude that HDL_3_, 22(R)-diol and pregnenolone promote CREB phosphorylation via upstream p38 MAPK signaling pathway. To provide additional evidence for this conclusion, we treated cells with these three agents in the presence and absence of a specific p38 MAPK inhibitor, SB203580 and reassessed the phosphorylation status of both p38 MAPK and CREB proteins. The results presented in Fig. 4 demonstrate that co-treatment of cells with SB203580 blocked the ability of HDL_3_, 22(R)-diol and pregnenolone to stimulate the phosphorylation of both p38 MAP and CREB, further confirming the p38 MAPK catalyzed phosphorylation of CREB.

### Steroid intermediates repress CREB activity

**Fig. 5:**
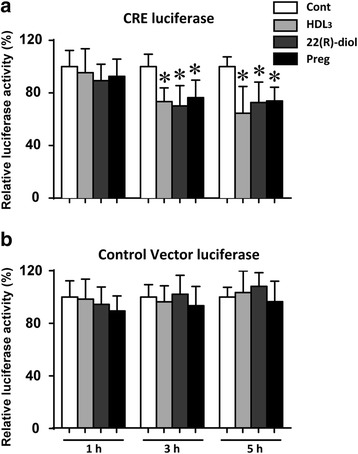
Steroid intermediates repress CRE reporter activity in Y1-BS1 cells. Y1-BS1 cells were transfected with Cre-Luc (firefly luciferase) (**a**) or pLuc-MCS vector (control) (**b**) and Renilla luciferase (Rluc, pRL-TK) for 24 h. Cells were then cultured in F-12K medium supplemented with 10% LPDS for 10 h before incubating with HDL_3_ (30 μg/ml), 22(R)-diol (10 μM) or pregnenolone (10 μM) for 1, 3 and 5 h followed by measurement of firefly and Renilla luciferase activities. Results are summary of three independent experiments with triplicates in each experiment. * *p* < 0.05

The above results indicate that phosphorylated forms of p38 MAPK and CREB were significantly increased in Y1-BS1 exposed to HDL_3_, 22(R)-diol or pregnenolone compared to vehicle control (Figs. 3 and 4). We next evaluated the impact of these agents on CREB activity using a CRE/CREB Reporter kit designed for monitoring the activity of the cAMP/PKA signaling in cultured cells. As shown in Fig. 5, CREB activity was slightly but significantly decreased in response to treatment with these three agents for 3 to 5 h. These data open up the possibility that overproduction/increased accumulation of steroid intermediates or sustained activation of cells to HDL leads to enhanced ROS production and consequently loss of CREB activity.

### Regulation of StAR and CYP11A1 gene expression

**Fig. 6:**
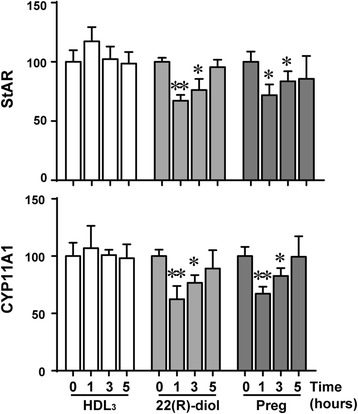
Down-regulation of StAR and CYP11A1 gene expression by steroid intermediates. Y1-BS1 cells were treated with or without HDL_3_ (30 μg/ml), 22(R)-diol (10 μM) or pregnenolone (10 μM) for different time. Cells were harvested, RNA isolated and reversed transcribed before subject to qRT-PCR analyses of StAR and CYP11A1 mRNA levels. 36B4 was used as internal control. Results are summary of three independent experiments with triplicates in each experiment. * *p* < 0.05, ** *p* < 0.01

StAR protein mediates rate-limiting step of steroidogenesis in which cholesterol was translocated from cytoplasm to mitochondria [46]. Then, P450scc (CYP11A1) catalyzes the conversion of StAR-delivered cholesterol to pregnenolone [47]. The transcription of both StAR and CYP11A1 is also regulated by tropic hormones involving cAMP signaling pathway in multiple transcription factors, including CREB [48]. Since we observed that CREB activity is repressed by HDL3, 22(R)-diol and pregnenolone, we measured the mRNA levels of its downstream targets, StAR and CYP11A1 following treatment of cells with these three agents. As shown in Fig. 6, the mRNA expression of both StAR and CYP11A1 was down-regulated by 22(R)-diol and pregnenolone treatment but not by hHDL_3_ for 1 or 3 h’ treatment, while the mRNA expression recovered after a longer time treatment.

### Inhibition of p38 MAPK phosphorylation upregulate steroidogenesis

**Fig. 7:**
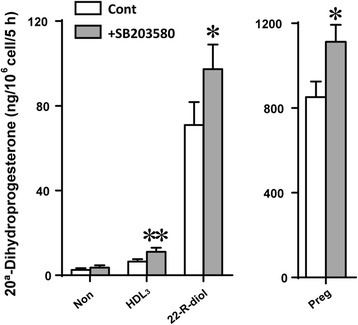
Inhibition of p38 MAPK activity by SB203580 augments steroid production. Y1-BS1 cells were pre-treated with or without SB203580 (10 μM) overnight. Cells were then incubated with HDL_3_ (30 μg/ml), 22(R)-diol (10 μM) or pregnenolone (10 μM) for 5 h. At the end of the incubation, the medium sampless were collected for hormone analysis using HPLC-MS / MS. Results are mean of three independent experiments with triplicates in each experiment. * *p* < 0.05, ** *p* < 0.01

We have demonstrated that inhibition of p38 MAPK activity with SB203580 potentiated the Bt_2_cAMP and Bt_2_cAMP + hHDL_3_-stimulated steroid production in Y1-BS1 adrenal cells [23]. Although, oxidants-induced activation p38 MAPK activity critically mediates the oxidant inhibition of steroid production, we also evaluated the potential effects of steroid metabolites and intermediates –induced activation of p38 MAPK on steroid synthesis. Y1-BS1 cells were treated with three steroid intermediates in the presence and absence of SB203580 and medium samples analyzed for steroid production using HPLC-MS/ MS. As shown in Fig. 7, although additional substrates (HDL_3_, 22(R)-diol or pregnenolone) loading increased the steroid production, inclusion of SB203580 to inhibit the p38 activity could increase the steroid production in HDL_3_, 22(R)-diol or pregnenolone treated cells. These findings are consistent with previous results showing that inhibition of p38 MAPK by SB203580 could ameliorate the oxidative stress-induced repression of steroidogenesis [23].

### Upregulation of anti-oxidant enzymes gene expression

**Fig. 8:**
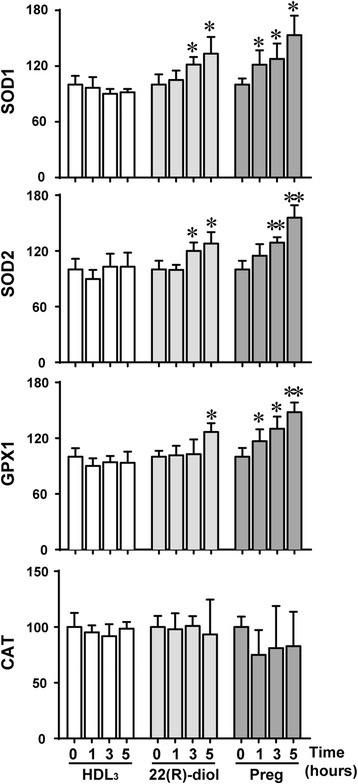
Up-regulation of anti-oxidant enzymes gene expression by steroid intermediates. Y1-BS1 cells were treated with or without HDL_3_ (30 μg/ml), 22(R)-diol (10 μM) or pregnenolone (10 μM) for various hours. RNA were isolated and reversed transcribed before subject to qRT-PCR analyses of SOD1, SOD2, GPX1 and CAT mRNA levels. 36B4 was used as internal control. Results are summary of three independent experiments with triplicates in each experiment. * *p* < 0.05, ** *p* < 0.01

ROS-induced oxidative stress is defined as a disturbance in the balance between production of ROS and antioxidants defenses, particularly the reduced functioning of the antioxidant enzymes (e.g., SOD1 [cytosolic CuZn-SOD], SOD2 [mitochondrial Mn-SOD], GPX1 [cytosolic/mitochondrial glutathione peroxidase 1], CAT [peroxisomal catalase] and PRDX3 [mitochondrial peroxiredoxin 3] [7, 8, 49–52]. Here we evaluated the mRNA expression levels of SOD1, SOD2, CAT and GPX1 expression in Y1-BS1 cells following their treatment with HDL_3_, 22(R)-diol or pregnenolone. Contrary to our expectation, we were surprised to see that 22(R)-diol and pregnenolone but not hHDL_3_ treatment induced the mRNA expression of SOD1, SOD2 and GPX (Fig. 8). In contrast, catalase expression was not affected by any of these treatments.

## Discussion

Steroidogenesis represents a highly complex and multistep metabolic process involving the participation of several catalytic enzymes, regulatory molecules and cellular organelles. More specifically, it involves the participation of various steroidogenic enzymes (e.g., CYP11A1, CYP11B1, 3βHSD), cholesterol transport proteins (e.g., SNAREs, StAR, vimentin), transcription factors (SF-1, DAX-1, AP-1, CREB and others) and availability of adequate amounts of substrate cholesterol [1, 2, 48, 53, 54]. Although tissue specific tropic hormones are the principal regulators of steroidogenesis, many growth factors, cytokines and pathophysiological conditions such as oxidative stress and ER stress also contribute to the regulation of steroidogenesis [1, 15, 53]. Because steroid synthesis involves many enzymatic steps, a number of intermediate steroid hormone metabolites are generated and sequentially utilized for the production of tissue specific steroid hormone. However, for many of these intermediates, conversion to subsequent metabolite is not efficient resulting in their increased accumulation in steroidogenic cells and as such could potentially impact steroidogenesis via increased ROS formation. Besides, steroidogenesis itself significantly contributes to the mitochondrial ROS production and in turn excessive oxidative stress can impede steroidogenesis [7, 8, 10, 23, 55]. As noted before, ROS are derived from molecular oxygen (O_2_) and comprise molecules with varying oxidant properties. At low concentrations, ROS modulate many cellular processes through redox-dependent signaling including proliferation, differentiation, apoptosis, immune regulation, cellular adaptation [11–13] and now it is becoming clear that they also participate in the (negative) regulation of steroidogenesis [7, 8, 10, 23, 55, 56]. In contrast, overproduction or high levels of ROS results in oxidative stress, a deleterious process that can be an important mediator of damage to cellular macromolecular structures including lipids and membranes, proteins and DNA [57, 58]. Thus, mammalian cells and more specifically, steroidogenic cells possess a potent anti-oxidant network to scavenge and neutralize the excessively produced ROS. Under normal physiological conditions, a balance between ROS production and elimination maintains optimal homeostasis. However, disturbances of such equilibrium under pathophysiological conditions can alter the functioning of normal cellular process with detrimental metabolic consequences.

In this study we made a novel observation that endogenous steroid intermediates such as 22(R)-diol and pregnenolone as well as a donor of cholesterol substrate, hHDL_3_ can promote ROS production and ensuing oxidative stress in Y1-BS1 adrenal cells. Our observations are in line with previous studies showing super oxide/ H_2_O_2_ produced during ACTH-stimulated adrenal steroid production is involved in the termination of the steroidogenic response [8]. They demonstrated that H_2_O_2_ produced by P450 enzymes during steroidogenesis inactivates potent mitochondrial antioxidant enzyme, peroxiredoxin 3, which in turn triggers a sequence of events including increased accumulation of H_2_O_2_, activation of p38 MAPK, suppression of StAR mRNA/protein expression and inhibition of steroidogenesis [8]. Our results suggest that it is not only the H_2_O_2_ produced during Mn-SOD catalyzed dismutation of superoxide anions and ensuing inactivation of peroxiredoxin 3 that inhibits steroidogenesis, but also steroid metabolites such as 22(R)-diol and pregnenolone possess similar capabilities in promoting ROS production and interfering with normal steroidogenesis. However, we were surprised to find that hHDL_3_ a cholesterol donor for steroidogenesis also promotes ROS production. This is even more surprising given that HDL is considered an antioxidant with relevance to cardiovascular disease [59]. At present we are unable to provide exact explanation by which HDL treatment of Y1 BS1 cells results in increased ROS production, but it may be that hHDL_3_ being a cholesterol donor, delivers excessive amounts of cholesterol to cells for its catabolism and this leads to exaggerated production of ROS and excessive oxidative stress. Obviously, more experimental studies are needed to sort out among these various possibilities.

Previous data suggest that hydrogen peroxide is a potent activator for p38 MAPK [23, 60]. Other reports indicate that tropic hormones such as FSH and ACTH also induce the phosphorylation and activation of p38 MAPK [8, 61]. Several investigators also reported that p38 MAPK is one of the regulators of CREB phosphorylation and its activity [22, 36, 62]. Our data provide evidence that HDL_3_, 22(R)-diol or pregnenolone treatment of Y1-BS1 results in robust phosphorylation of p38 MAPK as a consequence of enhanced ROS production. Under identical experimental conditions, the phosphorylation of one of the important steroidogenic transcription factors, CREB was also induced and based on the use of inhibitors, the observed phosphorylation of CREB is mediated by p38 MAPK. This observation is in agreement with previous reports showing that ROS upregulates CREB phosphorylation via p38 MAKP signaling cascade [22, 63]. Although cAMP-activated PKA and angiotensin II (AngII) activated protein kinase D (PKD) catalyzed phosphorylation of CREB at Ser133 is essential for CREB-induced gene transcription, the p38 MAPK-dependant phosphorylation of CREB have yielded conflicting results [48, 64]. Naqvi et al. reported that PKA catalyzed CREB phosphorylation and promoted the recruitment of the co-activator proteins CBP (CREB-binding protein) and p300 to increase the transcription of CREB-dependent genes in MEF cells, while MSK/MAPK also phosphorylated CREB without promoting recruitment of CBP or p300 and could not activated the transcription of CREB-dependent reporters [38]. A number of studies have reported that CREB is a target of p38 MAPK and that it phosphorylates and activates the activity of CREB [36, 62, 65]. On the contrary, other studies including our own have shown that p38 MAPK inhibits the transcriptional (reporter) activity of CREB in model steroidogenic cell lines, MLTC and Y1, while at the same time promotes CREB phosphorylation [22, 36, 62]. It is likely that these variable findings about p38 MAPK-mediated phosphorylation and altered CREB activity may be due to differences in cell types, metabolic conditions of the cell under study and functional interaction of CREB with specific co-activators/co-suppressors and/or other transcription factors. Our results provide evidence confirming previous findings from the laboratory that p38 MAPK inhibits steroidogenesis by attenuating CREB activity in a classic feedback mechanism.

Currently, we are exploring the potential mechanism by which ROS/p38 MAPK inhibition of CREB activity may lead to impaired steroidogenesis. One possibility we are considering is the inhibition of StAR gene transcription as a result of oxidant-mediated impaired CREB activity. This is in line with the observation that StAR expression is sensitive to both physiological and pathophysiological levels of ROS. Moreover, earlier studies have shown that CREB protein in cooperation with SF1 is a major regulator of StAR protein gene transcription [66, 67]. Finally, we have earlier shown that oxidants-p38 MAPK cause inhibition of StAR promoter activity primarily by interfering with CREB activity [22]. Other investigators reported that HDL_2_, very-low-density lipoprotein (VLDL) and glyco-oxidized VLDL can induce Cyp11B2 expression and stimulate steroid production in a human adrenocortical carcinoma cell line, NCI H295R [68–70]. Saha et al [70] also reported slight increases in StAR expression by native VLDL and glycol-oxidized VLDL but not by oxidized VLDL. The data presented here show that 22(R)-diol and pregnenolone but not HDL_3_ repress the gene expression of StAR and CYP11A1. We further provided the evidence that 22(R)-diol and pregnenolone-mediated repression of StAR and CYP11A1 gene expression is achieved through excessive oxidative stress and associated p38 MAPK signaling cascade. Our steroid hormone production data provide additional support to the notion that SB203580 inhibition of p38 MAPK augments the steroids production in Y1-BS1 cells treated with steroid metabolites/intermediates. A number of studies have implicated p38 MAPK signaling cascade in the regulation of steroidogenesis [71], although p38 MAPK regulation of steroidogenesis is complex and extent of p38 MAPK varies with steroidogenic cell types. For example, inhibition of p38 MAPK activity by SB203580 in IL-1α-stimulated immature rat Leydig cells leads to downregulation of StAR gene expression and attenuation of steroid production. [72]. Other studies have shown that inhibition of p38 MAPK activity by SB203580 in ovarian granulosa cells is accompanied by increased inhibition of LH/hCG/FSH mediated StAR expression and progesterone synthesis [61, 73]. Likewise, in primary cultures of rat adrenal glomerulosa cells, Angiotensin II activates the p38 MAPK and results in increases in StAR expression and steroid synthesis [71, 74]. Interestingly, our previous studies have shown that inhibition of p38 MAPK by either SB203580 or SB202190 in adrenocortical from old rats restores corticosterone synthesis to the levels seen in cells from young animals [23]. In this study, inhibition of p38 MAPK activity by SB203580 enhanced 20α-hydroxyprogesterone production in mouse Y1-BS1 adrenocortical tumor cell cotreated with HDL_3_, 22(R)-diol or pregnenolone. In addition, we will examine whether ROS/p38 MAPK modulate the expression of some of the critical SNARE proteins. In a recent publication, we identified several SNAREs, whose expression is essential for cholesterol transport to outer mitochondrial membrane for optimal steroid production [54].

Another surprising finding from our studies was that treatment of Y1-BS1 with 22(R)-diol and pregnenolone but not HDL_3_ leads to increased mRNA expression of three antioxidant enzymes, SOD1, SOD2 and CAT. In contrast, studies by Kil et al [8] observed no changes in the expression of levels of SOD1, SOD2, CAT, and GPX1 in intact adrenals following treatment of mice with ACTH. Interestingly, a recently published study reported that p38 MAPKα causes the induction of antioxidant enzymes SOD2 and catalase by two distinct mechanisms [18]. Obviously, more studies are needed to sort out molecular mechanisms involved in the transcriptional/posttranscriptional and/or posttranslational regulation of these antioxidant enzymes in vitro and in vivo.

## Conclusion

In conclusion, our studies provide evidence that exposure of adrenal cells to steroid intermediates/metabolites, 22(R)-diol and pregnenolone, and hHDL_3_ led to increased ROS production and associated enhanced phosphorylation of p38 MAPK and CREB. This oxidant-mediated up-regulation of CREB phosphorylation is mediated by p38 MAPK. The increased CREB phosphorylation however, was accompanied by a significant loss of CREB’s transcriptional activity. Furthermore, treatment of cells with 22(R)-diol and pregnenolone and ensuing oxidative stress resulted in decreased mRNA levels of StAR and CYP11A1 and increased mRNA levels of antioxidant enzymes SOD1, SOD2 and CAT. From these studies we conclude that ROS/p38 MAPK inhibition of CREB transcriptional activity is likely responsible for ROS-induced feedback inhibition of steroidogenesis.

## Abbreviations

22(R)-diol: 22(R)-Hydroxycholesterol
ACTH: Adrenocorticotropin hormone
AngII: Angiotensin II
CaM II: Calcium-calmodulin kinase II
CAT: Catalase
CREB: cAMP response element-binding protein
DCFH-DA: 2′,7′-Dichlorofluorescin diacetate
FBS: Fetal bovine serum
FSH: Follicle-stimulating hormone
GPX: Glutathione peroxidase
HDL: High-density lipoprotein
HNE: 4-hydroxy-2-nonenal
IMM: Inner mitochondrial membrane
LDL: Low-density lipoprotein
LH: Luteinizing hormone
LPDS: Lipoprotein-deficient serum
MSK: Mitogen- and stress-activated kinase
OMM: Outer mitochondrial membrane
PI3K: Phosphatidylinositol 3-kinase
PKA: Protein kinase A
PKB: Protein kinase B
PKC: Protein kinase C
PKD: Protein kinase D
pp90 RSK: pp 90 ribosomal S6 kinase
ROS: Reactive oxygen species
SF1: Steroidogenic factor 1
SOD: Superoxide dismutase
SR-BI: Scavenger Receptor Class B, Type I
VLDL: Very-low-density lipoprotein
